# Effect of drying and warming piglets at birth on preweaning mortality

**DOI:** 10.1093/tas/txab016

**Published:** 2021-02-04

**Authors:** Katherine D Vande Pol, Andres F Tolosa, Caleb M Shull, Catherine B Brown, Stephan A S Alencar, Michael Ellis

**Affiliations:** 1 Department of Animal Sciences, University of Illinois, Urbana–Champaign, IL 61801, USA; 2 The Maschhoffs, LLC, Carlyle, IL 62231, USA; 3 Departamento de Zootecnia, Federal University of Mato Grosso do Sul, Campo Grande, MS 79070-900, Brazil

**Keywords:** drying, farrowing, piglet, preweaning mortality, rectal temperature, warming

## Abstract

Piglets are susceptible to hypothermia early after birth, which is a major predisposing factor for preweaning mortality (**PWM**). Drying and warming piglets at birth has been shown to reduce early postnatal temperature decline. This study evaluated the effect of drying and warming piglets at birth on PWM and weaning weight (**WW**) under commercial conditions. A completely randomized design was used with 802 sows/litters (10,327 piglets); sows/litters were randomly allotted at start of farrowing to one of two Intervention Treatments (applied at birth): Control (no drying or warming); Drying+Warming (dried with a cellulose-based desiccant and placed in a box under a heat lamp for 30 min). Piglets were weighed at birth and weaning; PWM was recorded. Rectal temperature was measured at 0 and 30 min after birth on all piglets in a subsample of 10% of litters. The effect of farrowing pen temperature (**FPT**) on WW and PWM was evaluated by comparing litters born under COOL (<25°C) to those born under WARM (≥25°C) FPT. The effect of birth weight on WW and PWM was evaluated by comparing three birth weight categories (**BWC**; Light: <1.0 kg, Medium: 1.0 to 1.5 kg, or Heavy: >1.5 kg). PROC GLIMMIX and MIXED of SAS were used to analyze mortality and other data, respectively. Litter was the experimental unit; piglet was a subsample of litter. The model included fixed effects of Intervention Treatment, and FPT or BWC as appropriate, the interaction, and the random effects of litter. Piglet rectal temperature at 30 min after birth was greater (*P* ≤ 0.05) for the Drying+Warming than the Control treatment (+2.33°C). Overall, there was no effect (*P* > 0.05) of Intervention Treatment on PWM or WW, and there were no Intervention Treatment by BWC interactions (*P* > 0.05) for these measurements. There was an Intervention Treatment by FPT interaction (*P* ≤ 0.05) for PWM. Drying and warming piglets reduced (*P* ≤ 0.05) PWM under COOL (by 2.4 percentage units) but not WARM FPT. In addition, WW were lower (*P* ≤ 0.05) under WARM (by 0.79 kg) than COOL FPT; however, there was no interaction (*P* > 0.05) with Intervention Treatment. In conclusion, this study suggests that drying and warming piglets at birth increases rectal temperature and may reduce PWM under cooler conditions, which are typically experienced in temperate climates during the majority of the year.

## INTRODUCTION

On commercial swine units, the majority of preweaning mortality (**PWM**) of piglets occurs within the first 3 d of birth (Dyck and Swierstra, 1987; Su et al., 2007; KilBride et al., 2012), with crushing and starvation being the two most common causes of PWM (Dyck and Swierstra, 1987; Marchant et al., 2000). Hypothermia is often a major predisposing factor for both of these causes (Edwards, 2002). At birth, piglets have limited body surface insulation, a high body surface to volume ratio, and limited capacity for thermoregulatory heat production, resulting in a high critical temperature (around 35°C; Mount, 1959). In commercial practice, farrowing rooms are typically kept at temperatures between 20 and 22°C on the day of farrowing to prevent heat stress for the sows (PIC, 2018). Consequently, piglets are born into a relatively cool environment, resulting in considerable heat loss from the body surface due to convection and radiation. In addition, piglets are born wet and experience heat loss due to evaporation of the amniotic fluid. Therefore, without intervention, all piglets will experience some degree of body temperature decline immediately after birth (Vande Pol, 2020; Vande Pol et al., 2020a,b). This predisposes piglets to mortality, directly due to hypothermia as a primary cause and from secondary causes such as starvation, crushing, and disease (Devillers et al., 2011). Low-birth-weight piglets (i.e., those weighing < 1 kg) are particularly at risk of hypothermia and have the greatest rates of PWM (Herpin et al., 2002). Reducing the incidence of hypothermia early after birth should, therefore, decrease PWM, particularly in low-birth-weight piglets.

One common method of limiting piglet heat loss without increasing farrowing room temperature is to include a localized area in the farrowing pen with a higher temperature (e.g., with a heat lamp). However, piglets are generally not confined to this heated area, and are often more attracted to the sow in the early postnatal period (Houbak et al., 2006; Pedersen et al., 2006). Warming boxes (a box placed under a heat source) can be utilized to confine piglets for short periods of time after birth (typically 15 to 30 min) to minimize heat loss. Another method to reduce piglet heat loss is to limit evaporation by drying piglets at birth. Vande Pol et al. (2020b) showed that drying piglets with a desiccant at birth or confining them to a warming box for 30 min after birth were equally effective at reducing early postnatal temperature decline. However, the combination of these two approaches was more effective than using either one separately. Although both drying and warming of piglets early after birth are used in commercial practice, there has been limited published research on the effects of these approaches, either individually or in combination, on body temperature changes after birth and on PWM or weaning weight (**WW**). The objective of this study was to evaluate the effect of drying and warming newborn piglets on postnatal temperature changes and on piglet preweaning growth and mortality.

## MATERIALS AND METHODS

This study was conducted in the farrowing facilities of a commercial breed-to-wean farm of the Maschhoffs, LLC, located near Crawfordsville, IN, during the months of April–November 2018. The experimental protocol was approved by the University of Illinois Institutional Animal Care and Use Committee prior to the initiation of the research.

### Animals, Experimental Design, Treatments, and Allotment

A total of 402 sows and litters (10,327 piglets) were used in the study. Sows were from commercial dam lines of Yorkshire and Landrace origin that had been mated to commercial sire lines. The study used a completely randomized design, with litter as the experimental unit and piglet as a subsample of the litter, to compare two Intervention Treatments (applied at birth): Control (no drying or warming); Drying+Warming (piglets were dried at birth by coating with a commercial cellulose-based desiccant until completely dry, then placed in a plastic box under a heat lamp for 30 min; temperature in the box was 36.7 ± 3.12°C). Sows/litters were randomly allotted to Intervention Treatment at the start of farrowing (after the birth of the first piglet), with the restriction that dam genotype and parity were balanced across treatments.

### Housing and Management

Each sow was housed in an individual farrowing crate, located in the center of a farrowing pen, which had either woven metal or perforated plastic flooring. Crate dimensions were 0.55 m wide by 1.95 m long, giving a floor space within the crate of 1.07 m^2^; pen dimensions were 1.52 m wide by 2.07 m long, giving a total pen floor space of 3.15 m^2^. Crates were equipped with a sow-operated feed dispenser attached to a feed trough, and a nipple-type water drinker for the sow. An infrared heat lamp was suspended in the center of the floor area on one side of the farrowing crate over an insulated rubber mat (average temperature under the heat lamp during the study period was 37.1 ± 3.22°C). For the Drying+Warming treatment, the heat lamp was suspended over a plastic box for the duration of farrowing. Room temperature was maintained using fans, heaters, and evaporative coolers as needed; the thermostat for each room was set at 22.5°C on the day of farrowing and was incrementally reduced to 18.0°C by weaning.

Management in the farrowing facility was according to unit protocols, which were generally in line with standard commercial practices. Sows that had not farrowed by day 116 of gestation were induced to farrow on the following day using Lutalyse (1 injection of 1 mL given at 0600 h; Zoetis, Parsippany, NJ); the identity of each sow that was induced and date of induction were recorded. The farrowing process was monitored continuously by the investigators; if the interval between the births of piglets exceeded 60 min, the investigator checked the birth canal for obstructions and assisted the farrowing process as needed.

### Procedures and Measurements

At birth, piglets were given a uniquely numbered ear tag for identification, the allotted Intervention Treatment was applied, and they were returned to the farrowing pen (immediately for the Control and after 30 min in a warming box for the Drying+Warming treatment). Piglets were weighed within 12 h of birth using a Brecknell LPS-15 bench scale (Avery Weigh-Tronix, Fairmont, MN). Scales were calibrated daily prior to use with a standard test weight.

Piglet rectal temperature was measured at 0 and 30 min after birth on a randomly selected subsample of 10% of the litters distributed throughout the study period (41 litters and 527 live-born piglets on the Control treatment; 44 litters and 542 live-born piglets on the Drying+Warming treatment). Rectal temperatures were measured on all sows at the start and end of the farrowing process. Piglet and sow rectal temperatures were measured at a depth of 2.5 and 10 cm, respectively, using a HSTC-TT-K-24S-36 thermocouple attached via a SMPW-K-M connector to a dual input K/J digital thermometer (HH801A; Omega, Stamford, CT). Thermometers were calibrated each week during the study period by taking measurements in a temperature-controlled chamber that was set at temperatures that encompassed the expected range (i.e., 30, 32, 34, 36, 38, and 40°C). A regression equation for the relationship between measured and set temperatures was developed and was used to adjust rectal temperature measurements taken during the following week of the study.

The temperature in each farrowing pen at three locations [behind and at either side of the sow (one of these measurements being under the heat lamp)] was measured at the beginning and end of the farrowing process using a digital infrared thermometer [TOOGOO GM320 LCD digital infrared thermometer gun (Shenzhen IMC Digital Technology Co. Shenzhen, China)].

### Statistical Analysis

The litter of piglets was the experimental unit for all measurements; piglet was a subsample of litter. The PROC UNIVARIATE procedure of SAS (SAS Inst. Inc., Cary, NC) was used to verify normality and homogeneity of variances of the residuals. All variables that conformed to the assumptions of normality and homogeneity were analyzed using PROC MIXED of SAS (Littell et al., 1996). Preweaning mortality (PWM) data were analyzed using PROC GLIMMIX. The study was carried out using a completely randomized design; the model used for the analysis of sow and litter measurements accounted for the fixed effect of Intervention Treatment. The model used for analysis of Intervention Treatment differences in piglet weight, temperature, and PWM also included the random effect of litter.

An analysis was carried out to determine whether the response to Intervention Treatment differed according to piglet birth weight. The data set was divided on the basis of piglet birth weight into Light (<1.0 kg), Medium (1.0 to 1.5 kg), or Heavy (>1.5 kg) Birth Weight Categories (**BWC**). The maximum birth weight for the Light category (i.e., 1.0 kg) represented that below which PWM increases substantially (Zotti et al., 2017). The minimum birth weight for the Heavy category (i.e., 1.5 kg) represented that above which PWM is generally unaffected by birth weight (Zotti et al., 2017).

The study was carried out over a 10-mo period that included the summer months when the environmental temperature was relatively high. Consequently, during these periods, farrowing room temperatures were also relatively high and above the thermostat set point. This provided an opportunity to investigate the potential effect of ambient temperature in the farrowing rooms on piglet responses to drying and warming. The data set was divided on the basis of farrowing pen temperature on the day of farrowing into litters born under COOL (<25°C) or WARM (≥25°C) farrowing pen temperatures (**FPT**). The division at 25°C was chosen based on previous studies that suggested that piglet rectal temperatures are higher above this point compared with lower, more typical farrowing room temperatures (e.g., 20°C; Pedersen et al., 2013).

Piglet rectal temperature, WW, and PWM data were analyzed using a statistical model that included the fixed effects of Intervention Treatment, BWC or FPT, as appropriate, and the interaction, and the random effect of litter. For all analyses, differences between least-squares means were separated using the PDIFF option of SAS, and differences were considered significant at *P* ≤ 0.05. All *P*-values were adjusted using a Tukey’s adjustment for multiple comparisons.

## RESULTS AND DISCUSSION

Sow parameters and farrowing pen temperatures have been summarized by Intervention Treatment in Table 1. There were no differences (*P* > 0.05) between treatments for any of these except for temperature under the heat lamp before farrowing, which was greater (*P* ≤ 0.05) for the Control than the Drying+Warming treatment; however, this difference was relatively small (0.8°C). In general, the pigs used and temperature conditions in the farrowing facilities were typical of U.S. commercial production. The majority of sows on the study were between parities 1 and 8. Average sow rectal temperatures before and after farrowing were between 38.28 and 38.70°C, which is typical for farrowing sows (Littledike et al., 1979). Average farrowing pen temperatures (between 24.45 and 26.38°C; Table 1) were higher than the set point (22.5°C). This was expected; the study was conducted from April through November, which included the summer months, when it was difficult to reduce farrowing room temperatures.

**Table 1.** Summary of sow parity and rectal temperature and ambient temperatures in the farrowing pen during the study by Intervention Treatment

	Intervention Treatment^1^			
Item	Control	Drying+ Warming	SEM	*P*-value
Number of litters	400	402	—	—
Average sow parity^2^	4.1	4.1	0.14	0.96
Sow rectal temperature, °C				
Before farrowing	38.28	38.34	0.043	0.32
After farrowing	38.70	38.65	0.032	0.26
Farrowing pen temperature, °C				
Before farrowing				
Under heat lamp	37.14^a^	36.34^b^	0.149	0.0002
Side of pen opposite heat lamp	24.73	24.79	0.117	0.70
Behind sow	24.45	24.47	0.118	0.91
After farrowing				
Under heat lamp	37.24	37.55	0.158	0.18
Side of pen opposite heat lamp	26.13	26.38	0.136	0.20
Behind sow	25.62	25.73	0.137	0.58

^1^Control = piglets were not dried; Drying+Warming = piglets were dried at birth by coating with a desiccant, then placed in a warming box for 30 min.

^2^Parity = total number of litters including the one used in the study.

^a,b^Within a row, means with differing superscripts differ at *P* ≤ 0.05.

Numbers of litters and piglets, litter sizes, and piglet birth weights for the entire data set and for the subsample of 10% of litters used to measure piglet rectal temperatures are presented in Table 2. Number of piglets born alive and birth weights were similar (*P* > 0.05) for the Intervention Treatments for both the entire dataset and the subsample. In addition, there were no differences between Intervention Treatments (*P* > 0.05) for either litter size or birth weight between BWC treatments or between FPT treatments for the entire dataset or the subsample (Table 2). These results suggest that the subsample of litters used to measure piglet temperature was representative of the entire population. In addition, numbers born alive and birth weights were comparable to those reported for commercial swine populations at the time this study was conducted (PigChamp, 2018; Feldpausch et al., 2019; Vande Pol, 2020; Vande Pol et al., 2020a,b).

**Table 2.** Least-squares means for the effect of Intervention Treatment on litter size and piglet birth weight overall, and within Farrowing Pen Temperature (FPT)^2^ and Birth Weight Category (BWC)^3^ for the entire dataset and the subsample of 10% of litters

	Entire data set				Subsample			
	Intervention Treatment^1^				Intervention Treatment^1^			
Item	Control	Drying+ Warming	SEM	*P*-value	Control	Drying+ Warming	SEM	*P*-value
Number of litters	400	402	—	—	41	44	—	—
Number of piglets bornalive	5,164	5,163	—	—	527	542	—	—
By FPT^2^								
COOL	1,891	1,828	—	—	173	168	—	—
WARM	3,273	3,335	—	—	354	374	—	—
By BWC^3^								
Light	628	669	—	—	56	84	—	—
Medium	2,187	2,139	—	—	228	224	—	—
Heavy	2,349	2,355	—	—	243	234	—	—
Litter size, born alive								
Overall	12.9	12.7	0.19	0.55	13.4	12.2	0.57	0.13
By FPT^2^								
COOL	12.7	12.0	0.21	0.08	13.9	11.3	0.66	0.06
WARM	13.0	13.4	0.17	0.22	13.0	13.0	0.49	0.97
Piglet birth weight, kg								
Overall	1.49	1.48	0.013	0.67	1.49	1.43	0.042	0.34
By FPT^2^								
COOL	1.48	1.49	0.014	0.64	1.42	1.40	0.035	0.79
WARM	1.51	1.47	0.011	0.09	1.55	1.46	0.049	0.21
By BWC^3^								
Light	0.86	0.86	0.006	0.95	0.82	0.85	0.019	0.37
Medium	1.31	1.32	0.005	0.68	1.30	1.31	0.013	0.70
Heavy	1.78	1.77	0.004	0.17	1.79	1.74	0.013	0.06

^1^Control = piglets were not dried; Drying+Warming = piglets were dried at birth by coating with a desiccant, then placed in a warming box for 30 min.

^2^COOL < 25°C; WARM ≥ 25°C.

^3^Light < 1.0 kg; Medium = 1.0 to 1.5 kg; Heavy > 1.5 kg.

### Effect of Intervention Treatment

Least-squares means for the effect of Intervention Treatment on piglet rectal temperature at birth and 30 min after birth for the subsample of 10% of litters, and for WW and PWM for all litters in the study are presented in Table 3. Rectal temperatures at birth were similar (*P* > 0.05) for the two Intervention Treatments, which was expected as treatments were not applied until after birth temperature was measured. However, temperatures at 30 min after birth were 2.33°C lower (*P* ≤ 0.05; Table 3) for the Control than the Drying+Warming treatment. Vande Pol (2020) and Vande Pol et al. (2020b), in two studies that utilized the same Intervention Treatments and were carried out in the same facility as the current study, also found that temperatures at 30 min after birth were higher for piglets that had been dried and warmed at birth compared with untreated piglets. However, the magnitude of treatment difference was greater in the study of Vande Pol et al. (2020b; 2.9°C) than in the study of Vande Pol (2020; 1.6°C). The authors suggested that this difference in the magnitude of the response to drying and warming was most likely due to differences in temperatures in the farrowing facilities between these two studies (21.8 and 26.6°C, respectively). In support of this concept, farrowing pen temperatures in the current study averaged 25.4°C and the difference between the Intervention Treatments for piglet temperature at 30 min after birth was 2.33°C, which was intermediate to the treatment difference found in the two studies of Vande Pol (2020) and Vande Pol et al. (2020b).

**Table 3.** Least-squares means for the effect of Intervention Treatment (IT) on piglet weaning weight and preweaning mortality for the entire dataset, and on piglet rectal temperature at birth and 30 min after birth for the subsample of 10% of litters

	IT^1^			
Item	Control	Drying+ Warming	SEM	*P*-value
Piglet rectal temperature at birth, °C	38.72	38.65	0.051	0.38
Piglet rectal temperature at 30 min after birth, °C	35.65^b^	37.98^a^	0.095	<0.0001
Weaning weight, kg	5.35	5.23	0.053	0.07
Preweaning mortality, %	16.4	15.7	—	0.32
Age of piglets at death, d^2^	3.73	3.87	—	0.15

^1^Control = piglets were not dried; Drying+Warming = piglets were dried at birth by coating with a desiccant, then placed in a warming box for 30 min.

^2^Data were transformed using a square root prior to analysis to correct for normality and homogeneity of variance of the residuals.

^a,b^Within a row, means with differing superscripts differ at *P* ≤ 0.05.

There was no effect (*P* > 0.05) of drying and warming of piglets on WW, PWM, or the age of piglets at death (Table 3). This finding was unexpected given the positive effect of the Drying+Warming treatment on piglet temperatures at 30 min after birth discussed above. Low body temperature early after birth has been associated with an increased risk of mortality in a number of studies (e.g., Tuchscherer et al., 2000; Panzardi et al., 2013; Muns et al., 2016b); however, those studies were based on surveys of piglet traits associated with survival and did not include any intervention treatments. Relatively few studies have directly evaluated the effects of drying and/or warming of piglets at birth on preweaning growth or mortality, and these have produced variable results. Christison et al. (1997) found that PWM was lower for piglets that were either dried or warmed compared with an untreated control; however, there was no effect of these interventions on piglet WW. Andersen et al. (2009) found that piglets that were dried and/or placed under a heat lamp at birth had reduced PWM compared with untreated piglets; WW was not reported. In contrast, and in agreement with the results of the current study, a number of studies have reported that drying or warming piglets at birth had no effect on WW or PWM (McGinnis et al., 1981; Ogunbameru et al., 1991; Vasdal et al., 2011). Other studies have included drying or warming in combination with a number of other treatments, making it impossible to determine which factors caused any effects (White et al., 1996; Dewey et al., 2008). The PWM levels observed in the current study (around 16%) were marginally higher than average values reported for U.S. producers at the time this study was conducted (14.7% and 14.9%; PigChamp, 2017 and 2018, respectively). Further research is needed to clearly establish the effect, if any, of drying and warming of piglets at birth on performance to weaning, and also to determine whether these effects differ for farms with higher or lower PWM levels.

### Interactions Between Intervention Treatment and Farrowing Pen Temperature

Least-squares means for the effect of FPT and the interactions with Intervention Treatment for piglet rectal temperature for the subsample of litters, and for WW and PWM for all litters are presented in Table 4. Room temperatures were measured on each litter on the day of farrowing. The thermostat in each of the farrowing rooms used in this study was set at 22.5°C on the day of farrowing and, subsequently, was incrementally reduced to 18.0°C by weaning. Therefore, it could be argued that the temperature on the day of farrowing was not representative of that which persisted throughout lactation. However, the farrowing days with the higher pen temperatures corresponded to the summer months when cooling to the set point was not achieved and pen temperatures were consistently above 25°C, the temperature used to separate the two FPT treatments. In addition, allotments to the study were carried out on most days during the study period and the temperatures on consecutive days were relatively similar to those on the day of allotment. On this basis, the temperature on the day of farrowing was indicative of the conditions experienced throughout lactation.

**Table 4.** Least-squares means for the effect of farrowing pen temperature (FPT) and Intervention Treatment (IT) on piglet weaning weight and preweaning mortality for the entire dataset, and on piglet rectal temperature at birth and 30 min after birth for the subsample of 10% of litters

	FPT^1^				IT^2^ x FPT interaction	
Item	COOL	WARM	SEM	*P*-value	SEM	*P*-value
Piglet rectal temperature at birth, °C	38.57^b^	38.74^a^	0.052	0.03	0.053	0.25
Piglet rectal temperature at 30 min after birth, °C	—	—	0.094	0.01	0.094	0.03
Control	35.32^c^	35.98^b^	—	—	—	—
Drying+Warming	37.94^a^	38.03^a^	—	—	—	—
Weaning weight, kg	5.77^a^	4.98^b^	0.052	<0.0001	0.052	0.25
Preweaning mortality, %	—	—	—	0.93	—	0.05
Control	17.2^a^	15.9^ab^	—	—	—	—
Drying+Warming	14.8^b^	16.2^ab^	—	—	—	—
Age of piglets at death, d^3^	3.84	3.76	—	0.08	—	0.22

^1^COOL < 25°C; WARM ≥ 25°C.

^2^Control = piglets were not dried; Drying+Warming = piglets were dried at birth by coating with a desiccant, then placed in a warming box for 30 min.

^3^Data were transformed using a square root prior to analysis to correct for normality and homogeneity of variance of the residuals.

^a,b,c^Within a row (main effects), or interaction (if significant), means with differing superscripts differ at *P* ≤ 0.05.

Piglet temperatures at birth were greater (*P* ≤ 0.05) under WARM than COOL FPT; however, this difference was relatively small (<0.2°C). The body temperature of sows during farrowing has been shown to be higher under warmer than under cooler conditions (Muns et al., 2016a), which may be the cause of the difference in piglet birth temperature observed in the current study. There was an interaction (*P* ≤ 0.05) between FPT and Intervention Treatment for piglet temperature at 30 min after birth (Table 4). Temperatures of Control piglets were greater (*P* ≤ 0.05) under WARM than COOL FPT; in contrast, temperatures of piglets on the Drying+Warming treatment were similar (*P* > 0.05) for the two FPT. This resulted in the Drying+Warming treatment producing a greater increase in piglet temperature under COOL than WARM conditions (2.62 vs. 2.05°C, respectively; Table 4). In agreement with this result, Vande Pol (2020) also showed that drying and warming piglets at birth resulted in a greater increase in temperatures in the early postnatal period relative to untreated piglets under cooler than warmer farrowing room temperatures. These results suggest that although this intervention was effective at moderating the rectal temperature of piglets in the early period after birth across the range of temperatures typically experienced in commercial production, it was more effective under cooler conditions.

Piglet WW was greater (*P* ≤ 0.05) under COOL than WARM FPT; however, there was no interaction (*P* > 0.05) with Intervention Treatment (Table 4). There has been limited research on the effect of ambient temperatures during lactation on piglet WW. Similar to the results of the current study, Stansbury et al. (1987) found that litter WW were greater at lower (18 or 25°C) compared higher (30°C) farrowing room temperatures. Pedersen et al. (2015) found an interaction between birth weight and room temperature for WW. Low-birth-weight piglets (10th percentile) had lower WW at room temperatures of 15°C than 25°C, whereas the opposite was the case for heavy birth weight piglets (90th percentile). In the current study, there was no interaction (*P* > 0.05) between FPT and BWC (data not reported), indicating that higher farrowing pen temperatures reduced WW to a similar extent for piglets of all BWC. Higher temperatures during lactation can reduce sow milk production (Black et al., 1993), which could potentially explain the differences between FPT treatments for piglet WW in the current study.

There was an Intervention Treatment by FPT interaction (*P* ≤ 0.05) for PWM (Table 4). The Drying+Warming treatment had lower (*P* ≤ 0.05) PWM than the Control under COOL, but not WARM FPT. In addition, the average age of piglets at death tended (*P* = 0.08) to be lower under WARM than COOL FPT; however, this difference was very small and there was no interaction (*P* > 0.05) with Intervention Treatment. Hypothermia has been shown to be an important predisposing factor for PWM, either directly or indirectly (Edwards, 2002; Devillers et al., 2011). A number of studies have shown that drying and warming of piglets at birth reduces the extent and duration of low body temperature in the early postnatal period (Vande Pol et al., 2020a,b). However, as previously discussed, these studies and the current experiment have also shown that drying and warming of piglets at birth was more effective at reducing the extent and duration of postnatal temperature decline under cooler than warmer conditions.

Collectively, these results suggest that lower postnatal decline in temperature experienced by piglets at the higher ambient temperatures in the farrowing facilities did not predispose them to PWM. Nevertheless, drying and warming of piglets at birth was effective at reducing PWM at temperatures that prevail in farrowing facilities for major periods of the year, certainly in temperate climates. The only study to report on the effects of farrowing room temperature on PWM was that of Stansbury et al. (1987) which found that the lowest mortality was in rooms kept at an intermediate temperature (25°C) compared those at lower or higher temperatures (18 and 30°C, respectively). However, no piglet intervention treatments were applied in that study. There is a need for further research, ideally designed to directly compare room temperature treatments, to clarify the relationships between ambient temperature, piglet intervention treatments, and PWM.

### Interactions Between Intervention Treatment and Birth Weight Category

**Figure 1.**
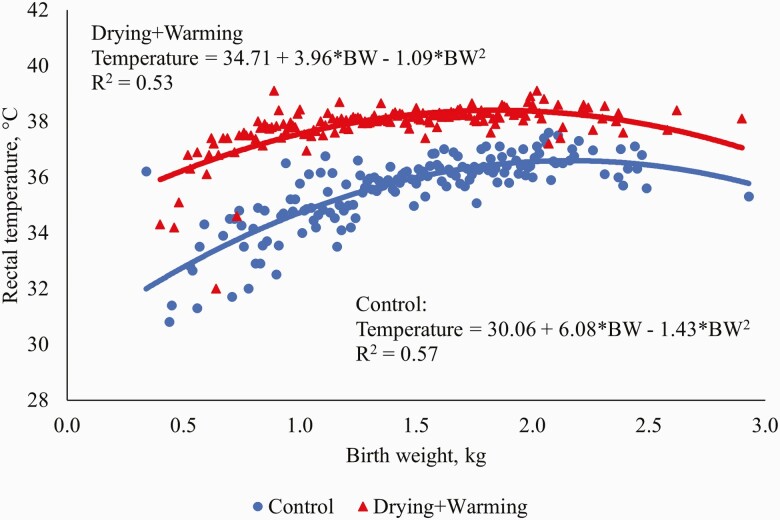
Regression relationships between piglet birth weight and rectal temperature at 30 min after birth for the Control and Drying+Warming treatments.

Least-squares means for the effect of piglet BWC and interactions with Intervention Treatment on piglet rectal temperature, WW, and PWM are presented in Table 5. Piglet temperatures at birth differed (*P* ≤ 0.05) between BWC; however, differences were small (<0.2°C). There was an interaction (*P* ≤ 0.05) between Intervention Treatment and BWC for piglet temperature at 30 min (Table 5). Light piglets had lower (*P* ≤ 0.05) temperatures than the other two BWC for both Intervention Treatments; however, this difference was greater for the Control than the Drying+Warming treatment. For example, the difference in temperature between Light and Heavy BWC was 2.49°C for the Control compared with 0.88°C for the Drying+Warming treatment. In addition, the Drying+Warming treatment resulted in greater (*P* ≤ 0.05) temperatures than the Control for all BWC, but the difference between the two treatments was greater for Light than Medium or Heavy piglets (3.49, 2.54, and 1.88°C higher, respectively; *P* ≤ 0.05). These results highlight that lighter birth weight piglets are more predisposed to hypothermia in the early postnatal period than heavier littermates, which is in agreement with the findings of a number of studies (Pattison et al., 1990; Pedersen et al., 2016; Cooper et al., 2019; Vande Pol, 2020; Vande Pol et al., 2020a,b). In addition, the results of the current study also suggest that drying and warming of piglets at birth was more effective at reducing the extent of postnatal temperature decline in lower birth weight piglets than for heavier littermates. This is illustrated by the regression relationships between piglet temperatures at 30 min after birth and birth weight for each Intervention Treatment, which are presented Figure 1. There was a quadratic relationship (*P* ≤ 0.05) between the two variables for both treatments; however, the relationships differed between treatments. Predicted temperatures were lower for the Control than for the Drying+Warming treatment for piglets of all birth weights (Figure 1); however, the change in temperature with increasing birth weight was greater for the Control than the Drying+Warming treatment. This is illustrated by the temperature difference between the lightest and heaviest birth weight piglets (i.e., 0.5 and 3.0 kg, respectively), which was relatively small for the Drying+Warming treatment (0.2°C) compared with the Control (2.5°C) treatment (Figure 1).

**Table 5.** Least-squares means for the effect of Birth Weight Category (BWC) and Intervention Treatment (IT) on piglet weaning weight and preweaning mortality for the entire data set and on piglet rectal temperature at birth and 30 min after birth for the subsample of 10% of litters

	BWC^1^					IT^2^ × BWC interaction	
Item	Light	Medium	Heavy	SEM	*P*-value	SEM	*P*-value
Piglet rectal temperature at birth, °C	38.56^c^	38.67^b^	38.73^a^	0.040	<0.0001	0.056	0.24
Piglet rectal temperature at 30 min after birth, °C	—	—	—	—	—	0.102	<0.0001
Control	33.83^e^	35.48^d^	36.32^c^	—	—	—	—
Drying+Warming	37.32^b^	38.02^a^	38.20^a^	—	—	—	—
Weaning weight, kg	3.73^c^	4.84^b^	5.86^a^	0.048	<0.0001	0.042	0.25
Preweaning mortality, %	44.6^a^	15.9^b^	8.2^c^	—	<0.0001	—	0.95
Age of piglets at death, d^3^	3.08^b^	4.27^a^	4.06^a^	—	<0.0001	—	0.19

^1^Light < 1.0 kg; Medium = 1.0 to 1.5 kg; Heavy > 1.5 kg.

^2^Control = piglets were not dried; Drying+Warming = piglets were dried at birth by coating with a desiccant, then placed in a warming box for 30 min.

^3^A square root transformation of the data was used prior to analysis to correct for normality and homogeneity of variance of the residuals.

^a,b,c,d,e^Within a row (main effects), or interaction (if significant), means with differing superscripts differ at *P* ≤ 0.05.

The regression relationships between piglet birth weight and rectal temperature at 30 min after birth for other studies that were carried out in the same facilities using the same Intervention Treatments as in the current study (Vande Pol, 2020; Vande Pol et al., 2020b) are presented in Table 6. Quadratic regression relationships gave the best fit to the data for both Intervention Treatments. Regression coefficients within each treatment were generally similar across all studies, indicating that the effect of piglet birth weight on rectal temperature was relatively consistent within each treatment. For all studies, intercepts were lower (*P* ≤ 0.05), and the linear and quadratic coefficients were greater (*P* ≤ 0.05) for the Control than the Drying+Warming treatment. These relationships further illustrate that drying and warming generally reduced the variation in piglet temperature due to birth weight, and resulted in lighter birth weight piglets having temperatures at 30 min after birth that were relatively similar to their heavier littermates.

**Table 6.** Regression coefficients for the linear and quadratic effects of piglet birth weight (BW) on piglet rectal temperature at 30 min after birth

	Control^1^				Drying+Warming^2^			
Study.	Intercept	BW	BW^2^	*R* ^2^	Intercept	BW	BW^2^	*R* ^2^
Current	30.06	6.08	−1.43	0.57	34.71	3.96	−1.09	0.53
Vande Pol et al. (2020b)	29.20	6.36	−1.44	0.36	35.45	3.17	−0.87	0.49
Vande Pol (2020)	30.54	6.85	−1.70	0.52	35.89	3.14	−0.82	0.45

^1^Control = piglets were not dried or warmed.

^2^Drying+Warming = piglets were dried at birth by coating with a desiccant, then placed in a warming box for 30 min.

Light piglets had lower (*P* ≤ 0.05) WW and higher (*P* ≤ 0.05) PWM than Heavy piglets; Medium piglets were intermediate and different (*P* ≤ 0.05) to the other BWC for both measurements (Table 5). A number of other studies have reported a negative relationship between birth weight and both WW and PWM (Charal, 2009; Panzardi et al., 2013). In addition, Quiniou et al. (2002) found that WW had a strong positive correlation with birth weight (*r* = 0.57). The average age of piglets at death was lower (*P* ≤ 0.05) for Light piglets compared with Medium or Heavy piglets, which were similar (*P* > 0.05); however, there was no interaction (*P* > 0.05) with Intervention Treatment for this measurement (Table 5). Le Dividich et al. (2017) also found that low-birth-weight piglets (with birth weights one SD below the mean or less) had a lower average age at death than heavier piglets (1.8 and 6.9 d, respectively). In addition, Vande Pol (2020) reported that the average age of piglets at death was lower for lighter than heavier birth weight piglets. These results highlight that piglet birth weight is a major factor influencing the preweaning performance of piglets.

Despite the considerable effect of birth weight on PWM observed in this study (Table 5), there was no interaction (*P* > 0.05) between Intervention Treatment and BWC for this measurement. This suggests that Drying+Warming was ineffective at reducing PWM compared with the Control in piglets of all birth weights (PWM of 43.7% and 45.5%, respectively, for Light piglets; 15.3 and 16.6%, respectively, for Medium piglets; 8.0 and 8.5%, respectively, for Heavy piglets). This result was unexpected given that this intervention reduced postnatal temperature decline to a greater extent for lower birth weight piglets than their heavier littermates (Table 5). Only one other study evaluated the effect of drying or warming on PWM for piglets of differing birth weights. Christison et al. (1997), in a small-scale study, found that drying with paper towels or placing piglets under a heat lamp reduced overall PWM for treated compared with untreated control piglets; however, this study was not able to detect treatment differences in PWM for low birth weight piglets (<1.05 kg), probably due to the low number of replications.

In the current study, the relative importance of piglet birth weight and postnatal temperature in determining the probability of PWM was evaluated using logistic regression analyses of the data from the subsample of piglets that had rectal temperature measurements taken, and the results of this analysis are presented in Table 7. Three different statistical models were used: Model 1 included piglet birth weight, Model 2 included piglet rectal temperature at 30 min after birth, and Model 3 included both factors. Quadratic terms tended to be significant (*P* ≤ 0.10) for both piglet birth weight and rectal temperature; therefore, linear and quadratic terms for these factors were included in the three models. Piglet birth weight (Model 1) and rectal temperature at 30 min after birth (Model 2) independently accounted for 74.3% and 62.5% of variation in PWM, respectively. However, including piglet rectal temperature and birth weight in the model (Model 3) only increased the variation in PWM explained to 74.6% (Table 7). This suggests that piglet birth weight was the most important factor for predicting PWM, and that, in comparison, piglet temperature at 30 min after birth was relatively unimportant.

**Table 7.** Regression coefficients for the linear effects of piglet birth weight (BW) and rectal temperature at 30 min after birth on the log odds of piglet preweaning mortality

Model^1^	Item	Coefficient	SE	*P*-value	OR^2^	ROC^3^
1						0.743
	Intercept	−1.99	0.115	<0.0001	—	
	BW	−2.33	0.294	<0.0001	0.10	
	BW^2^	0.77	0.497	0.10	2.17	
2						0.625
	Intercept	−1.79	0.102	<0.0001	—	
	30-min rectal temperature	−0.24	0.068	0.001	0.79	
	30-min rectal temperature^2^	0.04	0.024	0.09	0.10	
3						0.746
	Intercept	−1.98	0.123	<0.0001	—	
	BW	−2.19	0.303	<0.0001	0.11	
	BW^2^	0.60	0.51	0.24	1.82	
	30-min rectal temperature	−0.13	0.072	0.07	0.88	
	30-min rectal temperature^2^	−0.001	0.0235	0.98	1.00	

^1^Model 1 included linear and quadratic effects piglet birth weight; Model 2 included linear and quadratic effects of piglet rectal temperature at 30 min after birth; Model 3 included linear and quadratic effects of piglet birth weight and piglet rectal temperature at 30 min after birth.

^2^Odds ratio, values > 1 indicate an increase in the odds of mortality, values < 1 indicate a decrease in the odds of mortality.

^3^Receiver operating characteristic, a measure of variation in piglet preweaning mortality explained by the model.

A number of studies have carried out retrospective multivariate analyses of commercial data sets and have found that low birth weight is a major predisposing factor for PWM (Charal, 2009; Pedersen et al., 2011; Muns et al., 2016b). In addition, other studies have reported that low rectal temperature in the early postnatal period is a significant predisposing factor for PWM (Tuchscherer et al., 2000; Pedersen et al., 2011; Rothe, 2011; Muns et al., 2016b). However, the time of temperature measurement after birth that was most strongly related to mortality varied greatly across studies. Tuchscherer et al. (2000) suggested that piglet temperature at 1 h after had the strongest correlation with PWM (*r* = 0.22), whereas Muns et al. (2013) reported that temperature on the third day after birth was a better predictor than temperature measured on the first or second day. In addition, Panzardi et al. (2013) found that, of many factors evaluated, the odds ratio for PWM increased with decreasing birth weight and decreasing rectal temperature at 24 h after birth; however, birth weight explained substantially more variation in PWM than piglet rectal temperature. It needs to be emphasized that none of these studies utilized specific treatments, being based on analyses of population data from commercial facilities.

In conclusion, the results of this study found that drying and warming piglets at birth reduced piglet rectal temperature decline at 30 min after birth with differences relative to an untreated control that were consistent with those of previous research. However, there were no effects of drying and warming on WW or PWM, either overall or within any of the BWC. As expected, piglets of lower birth weight had lower WW and greater PWM than heavier littermates. Drying and warming piglets reduced PWM under cooler, but not warmer farrowing room temperatures. Preweaning mortality is complex, and postnatal change in piglet temperature is only one of a multitude of potential factors involved. There is a need for further large-scale controlled research studies to understand the potential role of piglet temperature changes and possible interactions with other factors in influencing piglet survival.
